# Supplementary material for: Monocytes from neonates and adults have a similar capacity to adapt their cytokine production after previous exposure to BCG and β-glucan
Source: PLoS One. 2020 Feb 21;15(2):e0229287. doi: 10.1371/journal.pone.0229287 (PMC7034882; doi:10.1371/journal.pone.0229287)
Supplement: Attachment — Submitted filename: NamakulaR_Responses_to_Reviewers_05Oct19.docx [file pone.0229287.s001.docx]

Professor Angelo A. Izzo	October 5^th^, 2019

Editor

PLoS ONE,

Dear Professor Izzo

**PONE-D-19-19049**

**Responses to Reviewer’s comments regarding the submitted manuscript entitled “Monocytes from neonates and adults have a similar capacity to elicit trained immunity responses to BCG”.**

*Thank you very much for the positive assessment and useful suggestions to our submitted manuscript. Please find responses to the Reviewer’s comments and changes in the manuscript have been effected.*

1. Please ensure that your manuscript meets PLOS ONE's style requirements, including those for file naming.

*We have revised the manuscript according to the PLOS ONE style templates as shown in the websites provided.*

2. Please provide additional details regarding participant consent. In the ethics statement in the Methods and online submission information, please ensure that you have specified whether consent was suitably informed. Since your study included minors under age 18, please ensure you have stated whether you obtained consent from parents or guardians in these cases.

*We obtained informed consent from the pregnant mothers. Eligibility requirements included pregnant mothers who were to deliver by cesarean section. The mothers were approached and the study purpose explained to them in a language they could understand. Those who gave informed consent on behalf of their newborn babies were recruited in the study. This has now been more clearly described in the revised manuscript.*

3. We note that you have stated that you will provide repository information for your data at acceptance. Should your manuscript be accepted for publication, we will hold it until you provide the relevant accession numbers or DOIs necessary to access your data. If you wish to make changes to your Data Availability statement, please describe these changes in your cover letter and we will update your Data Availability statement to reflect the information you provide.

*We shall provide the repository information for our data at acceptance.* *All data from the present study will be available as an Excel file from the NSD database (http://nsddata.nsd.uib.no with a doi:dx.doi.org/10.18712/NSD-NSD2722-V1), starting from the date of publication of this article.*

Additional Editor Comments:

As identified by the comments of the reviewers, there is too much ambiguity in the manuscript as it is written. There is a definite need to clarify the data and be more precise about the data and the related discussion. [Note: HTML markup is below. Please do not edit.]

*Thank you for this comment. In the revised version, we have been more precise about the data and the discussion.*

**Reviewers' comments:**

**Reviewer's Responses to Questions**

**Comments to the Author**

1. Is the manuscript technically sound, and do the data support the conclusions?

The manuscript must describe a technically sound piece of scientific research with data that supports the conclusions. Experiments must have been conducted rigorously, with appropriate controls, replication, and sample sizes. The conclusions must be drawn appropriately based on the data presented.

Reviewer #1: Partly

Reviewer #2: Yes

2. Has the statistical analysis been performed appropriately and rigorously?

Reviewer #1: Yes

Reviewer #2: Yes

3. Have the authors made all data underlying the findings in their manuscript fully available?

The PLOS Data policy<http://www.plosone.org/static/policies.action#sharing> requires authors to make all data underlying the findings described in their manuscript fully available without restriction, with rare exceptions (please refer to the Data Availability Statement in the manuscript PDF file). The data should be provided as part of the manuscript or its supporting information, or deposited to a public repository. For example, in addition to summary statistics, the data points behind means, medians and variance measures should be available. If there are restrictions on publicly sharing data—e.g. participant privacy or use of data from a third party—those must be specified.

Reviewer #1: Yes

Reviewer #2: No

4. Is the manuscript presented in an intelligible fashion and written in standard English?

PLOS ONE does not copyedit accepted manuscripts, so the language in submitted articles must be clear, correct, and unambiguous. Any typographical or grammatical errors should be corrected at revision, so please note any specific errors here.

Reviewer #1: Yes

Reviewer #2: Yes

5. Review Comments to the Author

Please use the space provided to explain your answers to the questions above. You may also include additional comments for the author, including concerns about dual publication, research ethics, or publication ethics. (Please upload your review as an attachment if it exceeds 20,000 characters)

Reviewer #1: This very basic study compared BCG and beta-glucan-induced cytokine expression by monocytes cultured from cord blood to monocytes from adult peripheral blood.

The cytokine expression by monocytes in response to BCG exposure was very modest and IL-6 was increased only. The title of this article states focuses only on the BCG response; however my opinion is that more data are required to convincingly show that these cord blood monocytes have been 'trained' (e.g. metabolic changes/epigenetic changes) beyond a very modest increase in a single cytokine.

Regarding the authors' conclusions: Are cord blood monocytes phenotypically equivalent to neonatal peripheral blood monocytes? The authors imply this is the case.

*We agree that we cannot be completely sure that cord blood monocytes are equivalent to neonatal monocytes. We did not identify any studies that have examined this question, which should be investigated in future studies. We have included a cautionary remark in the Discussion on Page 12 and paragraph 2 in the revised manuscript with track changes to acknowledge this.*

*Why were other cytokines not upregulated by BCG exposure (as in previous publications from the Netea group?)*

*We note that TNF and IL-10 cytokines did not show significant increase following BCG exposure in neither adults nor neonates in our study.*

*We, however, acknowledge that both IL-6 and TNF levels were increased in monocytes isolated from anonymous blood donors following BCG training in a similar in vitro assay in Kleinnijenhuis 2012 (1) and Bekkering 2016 (2). In the Bekkering paper, IL-6 responses were about 5 fold higher after 24h BCG training and later LPS restimulation, while TNF responses were about 2-fold higher. IL-10 responses did not reach significance. Encouraged by the above question we have from reviewers 1 and 2 we have added a paragraph in the manuscript discussing whether a less reactive BCG strain (BCG Bulgaria) was used in the present study. In both Kleinnijenhuis 2012 and Bekkering 2016, the BCG Denmark vaccine strain was used. Although it has not been tested for potential differences in inducing trained immunity, BCG Denmark has been shown to be more reactive and inducing higher cytokine levels upon homologous and heterologous stimulation (Anderson 2012, Ritz 2012) (3,4) This is likely the reason for weaker responses to BCG in both neonate and adults in our study, with only IL-6 being significant.*

*The reason for the use of BCG Bulgaria strain in the current study was represented by the production problems at SSI Denmark during the performance of the study, with BCG-Denmark strain not being available between 2016-2019.*

Had any of the adult volunteers been BCG vaccinated?

*For 27 adult volunteers vaccination status was known. 22 had been vaccinated, all of them many years ago, (until 2005 Norwegians were vaccinated at around age 10-14 years). 5 adults were not vaccinated. Responses in vaccinated and unvaccinated were generally similar, with the small group of 5 non-vaccinated tending to have slightly higher median responses than the vaccinated. With the long time since vaccination and similar responses in vaccinated and unvaccinated volunteers, we believe vaccination status is unlikely to have influenced results.*

Reviewer #2: Authors describe an experiment evaluating the cytokine produced after LPS stimulation adherent cells from cord blood and adult venous blood that were either non-primed or primed with BCG or B-glucan. Both BCG and B-glucan can "train" innate immune cells, essentially the response to a secondary stimulus (typically a TLR agonist) is increased in trained cells over non-trained cells. Much of the data to date has focused on adults, and maybe children, the ability of mononuclear cells (primarily monocytes) to undergo innate training has not been demonstrated. The use of cord blood provides one mechanism to evaluate neonate monocyte ability to undergo training.

Overall, the intent of the study was a single question, and with some improvements to writing, describes training potential of neonatal cells.

Comments:

More differences were observed with B-glucan than BCG, yet the title only mentions BCG, and discussion is focused on BCG. Inclusion of B-glucan into the title, abstract, intro, and discussion are warranted. The b-glucan findings are just as important. Some revision for verb tense is warranted. New data should be past tense, and established (ie, published data) is present tense.

*We agree. We have now included β-glucan in the title, abstract, intro and added discussion of these findings in the discussion as advised and the revision for verb tense has been done.*

Line 69 references children and adults, line 74 only refers to adults.

*Line 69 refers to some of the studies that have demonstrated trained innate immunity in both children and adults. However, line 74 describes the in vitro induction of trained immunity model which was studied in adults but not neonates, hence the reason for us to study this model in neonates.*

Is data from children the better comparison? Perhaps best to clearly define by age neonate to children?

*There is hardly data on trained innate immunity of adherent mononuclear cells in neonates.*

*We agree that it is better to change "children" to "infants" as shown in a few studies done in infants; Smith SG et al, 2017 (5) and Jensen KJ, et al, 2015 (6). These studies however, didn't use adherent monocytes and were post-vaccination studies.*

*To be more specific we have changed the word “children” to “infants” in the introduction.*

Line 116 – is it a company or a lab that provided the BCG? It only reads as TN, USA which is unclear.

*The reviewer here obviously meant β-glucan and not BCG. β-glucan was kindly provided by Professor David Williams (East Tennessee State University, USA). We have corrected this in the Methods section.*

Line 129 – define the negative control – you mean RPMI-only wells?

Same for Line 132

*Yes, the negative control is the RPMI- only wells. We have now defined it more clearly in the mentioned paragraphs.*

Graph titles may be better presented at top of graph, versus below. Can the cytokine name be moved to the top (as opposed to below?).

*We thank the reviewer for good suggestions. The figures have been improved accordingly.*

Line 148 – given how the data is presented (expressed as fold change) the use of the words “remained stable” are misleading. Perhaps it’s “were not different” instead, as remained stable suggests the cytokine was produced (and perhaps it was, but with normalization to RPMI-only group, that cannot be visualized.

*Yes, we agree that “were not different” is a clearer formulation. This has been corrected in the revised manuscript.*

Expand on line 150 to include name of all three cytokines.

*Naming of all three cytokines has been included.*

Line 144 and 161 warrant revision for clarity – the response isn’t to BCG or B-glucan, it’s to LPS after prior BCG or B-glucan exposure. Include the word training or expand to say “…to LPS following BCG or B-glucan priming”

*We agree. This has been corrected to include “to LPS following BCG or β-glucan priming”*

Line 170 – this means you did statistics between the adult and neonate for each respective treatment?

*Yes, to help make this clearer we rephrased sentences about comparison between adults and neonates.*

Line 181 – that data is not shown. The response to LPS would be in the RPMI only group or the RPMI+LPS group? As a reader, we cannot appreciate the response of CBAM to LPS because that was used as the normalizer. Perhaps it’s an important point in general if the same amount of IL6 is produced by non-trained cells after LPS stimulation. The sentence warrants revision for clarity.

*We agree that this sentence can be better formulated to reflect the presented data. The production of cytokines in the BCG and β-glucan primed cells that were restimulated with LPS were normalized against the cytokine production in the non-primed (RPMI only) LPS-restimulated cells. For more information: there was cytokine production in the non-primed cell cultures used for normalization, and this was similar in adults and neonates; i.e. for any of the three cytokines, measured levels of non-primed, LPS-restimulated monocytes were not significantly different in adult and neonates.*

*In the revised manuscript, the inserted sentence “and that CBAM respond to LPS re-stimulation” has been removed for clarity.*

This is also true for the following sentence into line 182 – CBAM response to LPS can be maintained following initial training – again, left to infer that cytokine was produced, but because of the normalization of the data, that is not apparent. We just know it wasn’t different between non-trained (RPMI only) and BCG primed (or trained) cells.

*We agree and have clarified the sentence to “Our data shows that some CBAM cytokine responses, when restimulated with LPS following initial training with either BCG or β-glucan stimuli, are increased compared to LPS-restimulated non-trained CBAM.” as this is what we want to show by calculating and presenting our data as fold change.*

Line 192 – can the authors try to explain why there may be a difference? It’s nice they can contrast to prior studies, but why is this study different? Was the source of BCG different between the two studies?

*We note that TNF and IL-10 cytokines did not show significant increase following BCG exposure in neither adults nor neonates in our study.*

*We, however, acknowledge that both IL-6 and TNF levels were increased in monocytes isolated from anonymous blood donors following BCG training in a similar in vitro assay in Kleinnijenhuis 2012 (1) and Bekkering 2016 (2). In the Bekkering paper, IL-6 responses were about 5 fold higher after 24h BCG training and later LPS restimulation, while TNF responses were about 2-fold higher. IL-10 responses did not reach significance. Encouraged by the above question we have from reviewers 1 and 2 we have added a paragraph in the manuscript discussing whether a less reactive BCG strain (BCG Bulgaria) was used in the present study. In both Kleinnijenhuis 2012 and Bekkering 2016, the BCG Denmark vaccine strain was used. Although it has not been tested for potential differences in inducing trained immunity, BCG Denmark has been shown to be more reactive and inducing higher cytokine levels upon homologous and heterologous stimulation (Anderson 2012, Ritz 2012) (3,4) This is likely the reason for weaker responses to BCG in both neonate and adults in our study, with only IL-6 being significant. The reason for the use of BCG Bulgaria strain in the current study was represented by the production problems at SSI Denmark during the performance of the study, with BCG-Denmark strain not being available between 2016-2019.*

*Another difference was use of different concentrations of BCG. In Kleinnijenhuis 2012 and Bekkering 2016 studies, they used 1µg/mL and 10µg/mL, respectively, while in the current study we used 5µg/mL.*

Line 210 warrants a citation or a profile of CBAM – what percentage of what remains in the dish is expected or shown to be other cell types that would not be present using adult venous blood? Most of the data coming from the mouse world shows such tight error bars and similar responses with training. The human data set has greater differences, acknowledgement in the discussion is warranted. Is this genetics, or exposure to immune altering compounds in utero?

*We agree that this is a fair discussion point. A profile of CBAM in a study by Normann Erik et al, showed a purification of non‐active, but stimulation‐competent monocytes with high yields (2.3–9×10^7^ cells) and purity of 70–90% (average 83%) by adherence or 90–98% by flow cytometric analysis (7).*

*We have added this issue and the reference in the Discussion.*

6. PLOS authors have the option to publish the peer review history of their article (what does this mean?<https://journals.plos.org/plosone/s/editorial-and-peer-review-process#loc-peer-review-history>). If published, this will include your full peer review and any attached files.

If you choose “no”, your identity will remain anonymous but your review may still be made public.

Do you want your identity to be public for this peer review? For information about this choice, including consent withdrawal, please see our Privacy Policy<https://www.plos.org/privacy-policy>.

Reviewer #1: No

Reviewer #2: No

Please log into your account, locate the manuscript record, and check for the action link "View Attachments". If this link does not appear, there are no attachment files to be viewed.]

While revising your submission, please upload your figure files to the Preflight Analysis and Conversion Engine (PACE) digital diagnostic tool, https://pacev2.apexcovantage.com/. PACE helps ensure that figures meet PLOS requirements. To use PACE, you must first register as a user. Registration is free. Then, login and navigate to the UPLOAD tab, where you will find detailed instructions on how to use the tool. If you encounter any issues or have any questions when using

PACE, please email us at figures@plos.org<mailto:figures@plos.org>.

Please note that Supporting Information files do not need this step.

References

1. *Kleinnijenhuis J, Quintin J, Preijers F, et al. Bacille Calmette-Guerin induces NOD2-dependent nonspecific protection from reinfection via epigenetic reprogramming of monocytes. Proc Natl Acad Sci U S A 2012; 109:17537-42.*
2. *Bekkering S, Blok BA, Joosten LA, Riksen NP, van Crevel R, Netea MG. In Vitro Experimental Model of Trained Innate Immunity in Human Primary Monocytes. Clin Vaccine Immunol 2016; 23:926-33.*
3. *Anderson EJ WE, Mawa PA, Kizza M, Lyadda N, Nampijja M, Elliott AM. The influence of BCG vaccine strain on mycobacteria-specific and non-specific immune responses in a prospective cohort of infants in Uganda. Vaccine. 2012;30(12):2083-9.*
4. *Ritz N, Dutta B, Donath S, Casalaz D, Connell TG, Tebruegge M, et al. The influence of bacille Calmette-Guerin vaccine strain on the immune response against tuberculosis: a randomized trial. Am J Respir Crit Care Med. 2012;185(2):213-22.*
5. *Smith SG, Kleinnijenhuis J, Netea MG, Dockrell HM. Whole Blood Profiling of Bacillus Calmette-Guerin-Induced Trained Innate Immunity in Infants Identifies Epidermal Growth Factor, IL-6, Platelet-Derived Growth Factor-AB/BB, and Natural Killer Cell Activation. Frontiers in immunology. 2017;8:644.*
6. *Jensen KJ, Larsen N, Biering-Sorensen S, Andersen A, Eriksen HB, Monteiro I, et al. Heterologous immunological effects of early BCG vaccination in low-birth-weight infants in Guinea-Bissau: a randomized-controlled trial. J Infect Dis. 2015;211(6):956-67.*
7. *Normann E, Lacaze-Masmonteil T, Winkler-Lowen B, Guilbert LJ. Isolation of non-activated monocytes from human umbilical cord blood. Am J Reprod Immunol 2010; 63:66-72.*
