# Supplementary material for: Monocytes from neonates and adults have a similar capacity to adapt their cytokine production after previous exposure to BCG and β-glucan
Source: PLoS One. 2020 Feb 21;15(2):e0229287. doi: 10.1371/journal.pone.0229287 (PMC7034882; doi:10.1371/journal.pone.0229287)
Supplement: Attachment — Submitted filename: Rebuttal letter 14Nov19.docx [file pone.0229287.s002.docx]

Professor Angelo A. Izzo	November 11^th^, 2019

Editor

PLoS ONE,

Dear Professor Izzo

**PONE-D-19-19049R1**

**Responses to Reviewer’s comments regarding the submitted manuscript entitled “Monocytes from neonates and adults have a similar capacity to elicit trained immunity responses to BCG”.**

*Thank you very much for the positive assessment to our revised submitted manuscript. Please find responses to the reviewer’s comments to our revised manuscript. The manuscript has been revised according to many of the comments. We thank the reviewers for taking the time to point out some grammatical improvements.*

Comments to the Author

1. If the authors have adequately addressed your comments raised in a previous round of review and you feel that this manuscript is now acceptable for publication, you may indicate that here to bypass the “Comments to the Author” section, enter your conflict of interest statement in the “Confidential to Editor” section, and submit your "Accept" recommendation.

Reviewer #1: (No Response)

Reviewer #2: All comments have been addressed

2. Is the manuscript technically sound, and do the data support the conclusions?

The manuscript must describe a technically sound piece of scientific research with data that supports the conclusions. Experiments must have been conducted rigorously, with appropriate controls, replication, and sample sizes. The conclusions must be drawn appropriately based on the data presented.

Reviewer #1: No

Reviewer #2: (No Response)

3. Has the statistical analysis been performed appropriately and rigorously?

Reviewer #1: Yes

Reviewer #2: (No Response)

4. Have the authors made all data underlying the findings in their manuscript fully available?

The PLOS Data policy<http://www.plosone.org/static/policies.action#sharing> requires authors to make all data underlying the findings described in their manuscript fully available without restriction, with rare exception (please refer to the Data Availability Statement in the manuscript PDF file). The data should be provided as part of the manuscript or its supporting information, or deposited to a public repository. For example, in addition to summary statistics, the data points behind means, medians and variance measures should be available. If there are restrictions on publicly sharing data—e.g. participant privacy or use of data from a third party—those must be specified.

Reviewer #1: Yes

Reviewer #2: (No Response)

5. Is the manuscript presented in an intelligible fashion and written in standard English?

PLOS ONE does not copyedit accepted manuscripts, so the language in submitted articles must be clear, correct, and unambiguous. Any typographical or grammatical errors should be corrected at revision, so please note any specific errors here.

Reviewer #1: Yes

Reviewer #2: (No Response)

6. Review Comments to the Author

Please use the space provided to explain your answers to the questions above. You may also include additional comments for the author, including concerns about dual publication, research ethics, or publication ethics. (Please upload your review as an attachment if it exceeds 20,000 characters)

Reviewer #1: My original major concern remains: I do not feel it is sufficient to term cytokine production by monocytes 'training' without showing additional features that are characteristic of innate immune training (eg switch in metabolism to glycolysis, epigenetic changes, other functional changes such as reactive oxygen species production or the longevity of the response). The authors have not addressed this concern and should do so by re-considering their aims and conclusions. The cytokine production may indicate in vitro training, but the authors do not provide any evidence that this was the case and need to acknowledge this.

Answer. This is an important point that is raised by the reviewer. Strictly, the term of trained immunity depicts the long-term capacity of innate immune cells to over-respond to a second stimulation, after the short-term exposure to an initial stimulus. The marker of that is generally used is cytokine production, while the mechanisms behind it are metabolic and epigenetic. This has been shown by measuring cytokines in a number of studies, and the in vitro model we use it has been described for adults.

We have now used the same model in cord blood monocytes. The experimental method used in this study is an in vitro model of trained immunity published in several papers (Kleinnijenhuis et al., 2012, Arts et al 2016 and Bekkering et al 2016). In addition to measuring cytokine responses as proof of training, the mentioned studies also used the model to investigate the metabolic and epigenetic mechanisms related to trained immunity. Although this would have been interesting as added measurements of trained immunity, our focus in the present study was to compare adult and cord blood monocyte trained immune responses in a well-established model with the commonly used immunological readouts of trained immunity. Not enough material is unfortunately currently available to extend these studies to metabolic and epigenetic parameters.

We have addressed the reviewers concern by stating in the discussion that epigenetic and metabolic changes to further define a ‘broad trained immunity phenotype’ was not done in this study. In the immunological sense, showing hyper-responsiveness is enough to demonstrate trained immunity.

The authors should include the vaccination status of the adult volunteers in the Methods.

Answer. We have now included vaccination status of adults in methods section of the revised manuscript.

Title: revisit - is this really training?

Answer. Based on the arguments exposed above, we argue that the process studied in this ex-vivo model is indeed training, both by definition and by readouts in an established model for trained immunity.

Abstract: L38 - this study does not address the ability of BCG and beta-glucan to induce trained immunity in neonates. It studies the ability of BCG and beta-glucan to induce cytokine production in cord blood monocytes.

Answer. In our opinion “the ability of BCG and beta-glucan to induce cytokine production in cord blood monocytes”, when restimulated with LPS after a resting period is per definition a trained immunity response as seen in this model in several studies and the way this term is used in the current literature. We do not merely study the direct responsiveness of cord blood monocytes to acute stimulation. We therefore believe the text in the abstract reflects this correctly.

However, we agree that replacing the term “neonates” with “cord blood monocytes” in this sentence states the aim in a more specific way, and this has been replaced in the abstract.

Typographical errors:

L55 - use the abbreviation TB

The abbreviation ‘TB’ has been used instead of ‘tuberculosis’ in the revised manuscript.

L154 females --> female

This error has been corrected.

L163 cytokine levels --> cytokines (IL-6, ...)

The cytokines (IL-6, IL-10 and TNF) have been mentioned in the revised manuscript.

L213 - replace the 'the'

‘the’ has been replaced in the manuscript

L247 - ... therefore this should be ...

The sentence has been revised in the manuscript

L248 The Results --> The results

This has been corrected in the manuscript

Tenses are still a bit confused in the discussion - established knowledge/data in the present tense, but when you refer to a specific study (published or not) use the past tense.

Thanks for this observation; tenses in the discussion have been revised once again in the revised manuscript

Reviewer #2: (No Response)

7. PLOS authors have the option to publish the peer review history of their article (what does this mean?<https://journals.plos.org/plosone/s/editorial-and-peer-review-process#loc-peer-review-history>). If published, this will include your full peer review and any attached files

If you choose “no”, your identity will remain anonymous but your review may still be made public.

Do you want your identity to be public for this peer review? For information about this choice, including consent withdrawal, please see our Privacy Policy<https://www.plos.org/privacy-policy>.

Reviewer #1: No

Reviewer #2: No

[NOTE: If reviewer comments were submitted as an attachment file, they will be attached to this email and accessible via the submission site. Please log into your account, locate the manuscript record, and check for the action link "View Attachments". If this link does not appear, there are no attachment files to be viewed.]

While revising your submission, please upload your figure files to the Preflight Analysis and Conversion Engine (PACE) digital diagnostic tool, https://pacev2.apexcovantage.com/. PACE helps ensure that figures meet PLOS requirements. To use PACE, you must first register as a user. Registration is free. Then, login and navigate to the UPLOAD tab, where you will find detailed instructions on how to use the tool. If you encounter any issues or have any questions when using PACE, please email us at figures@plos.org<mailto:figures@plos.org>. Please note that Supporting Information files do
