# Supplementary material for: Monocytes from neonates and adults have a similar capacity to adapt their cytokine production after previous exposure to BCG and β-glucan
Source: PLoS One. 2020 Feb 21;15(2):e0229287. doi: 10.1371/journal.pone.0229287 (PMC7034882; doi:10.1371/journal.pone.0229287)
Supplement: Attachment — Submitted filename: Rebuttal letter 23Des19.docx [file pone.0229287.s003.docx]

Professor Angelo A. Izzo	December 23^th^, 2019

Editor

PLoS ONE,

Dear Professor Izzo

**PONE-D-19-19049R2**

**Responses to comment from Reviewe#1 regarding the resubmitted manuscript entitled “Monocytes from neonates and adults have a similar capacity to elicit trained immunity responses to BCG”.**

Reviewer #1: The authors strongly argue that what they observe (increased cytokine production) is trained immunity. That may be true; however I still have concerns about this, not least because the responses are quite different (perhaps due to the BCG strain used) from previously published data. This could mean that the 'training' (ie epigenetic/metabolic changes etc) observed in the earlier published work did not occur here.

In order to feel satisfied accepting this I think the authors should restrain their conclusions (ie in the abstract instead of saying "BCG and B-glucan induce a similar level of trained immunity in neonatal monocytes" they should more carefully state "BCG and B-glucan induce increased cytokine production, reminiscent of trained immunity, in neonatal monocytes".

I understand it is not possible to do further testing on the samples; however simply measuring cytokines is insufficient to indicate trained immunity, especially when the results are different (diminished) compared to the published work using this model.

**Reply: We agree that we could be a little more cautious in the interpretation, and we have changed the relevant sentences in conclusions in the abstract and manuscript in line with the reviewer’s request.**

**To further underline the issue raised by the reviewer we also added the sentence “Including measurement of epigenetic and metabolic alterations in such experiments will be helpful to characterize the nature of the effects and mechanisms behind them“ in the discussion about further experiments.**
