# Supplementary material for: Monocytes from neonates and adults have a similar capacity to adapt their cytokine production after previous exposure to BCG and β-glucan
Source: PLoS One. 2020 Feb 21;15(2):e0229287. doi: 10.1371/journal.pone.0229287 (PMC7034882; doi:10.1371/journal.pone.0229287)
Supplement: Attachment — Submitted filename: Rebuttal letter 22Jan20.docx [file pone.0229287.s004.docx]

Professor Angelo A. Izzo	January 22^nd^, 2019

Editor

PLoS ONE,

Dear Professor Izzo

**PONE-D-19-19049R3**

**Responses to Reviewer’s comments regarding the submitted manuscript entitled “Monocytes from neonates and adults have a similar capacity to elicit trained immunity responses to BCG”.**

*Thank you very much for the further assessment to our revised submitted manuscript. Please find responses to the Reviewer’s comments and changes made in regards to trained immunity concept.*

Comments to the Author

Reviewer #1: The authors have made some quite minor amendments to the manuscript to address my concern, which is that I think it is incorrect to use the term 'trained immunity', without evidence of this beyond cytokine production particularly given that the cytokine responses observed in this study differed to those from the cited earlier publications that use the 'well-established' model.
This authors can address this by properly restraining conclusions in the title, abstract and full manuscript; however the authors have only made a small attempt to do this and in my opinion most people giving this manuscript a cursory read, as it stands, would not notice that other aspects of training (beyond cytokine production) have not been determined and it is relying on previous published work

**Response:**

**In the previous revision, we introduced the changed requested by the reviewer. When it is now requested to make more thorough revision of the use of the term “trained immunity” we have discussed this between us and acknowledge that there are different opinions regarding the use of this term.**

**In addition to the already introduced cautious wording “reminiscent of trained immunity”, as advised by the reviewer previously, we have therefore now revised the manuscript to focus on presenting the cytokine response data. We have used more cautious wording and made amendments to the revised manuscript with properly restraining conclusions regarding “trained immunity” in the title, abstract and full manuscript.**
